# Acute Fatty Liver in Pregnancy: Literature Review

**DOI:** 10.7759/cureus.94576

**Published:** 2025-10-14

**Authors:** Su Zarni, Amrita Viegas

**Affiliations:** 1 Obstetrics and Gynaecology, Luton and Dunstable Hospital, Luton, GBR; 2 Obstetrics, Luton and Dunstable Hospital, Luton, GBR

**Keywords:** aflp, icu care, liver failure, liver transplantation, maternal mortality, pregnancy

## Abstract

Acute fatty liver of pregnancy (AFLP) is a rare but potentially life-threatening obstetric emergency that typically arises in the third trimester. Although uncommon, its rapid onset, multisystem involvement, and historically high mortality make it an essential condition for clinicians to recognize and manage promptly. Its pathogenesis has been linked to defects in fatty acid metabolism and an abnormality in mitochondrial beta oxidation is a recognised cause of AFLP in a subset of cases.

The clinical significance of AFLP lies in its dramatic course and overlap with other hepatic disorders of pregnancy such as haemolysis, elevated liver enzymes and low platelets (HELLP) syndrome, preeclampsia, and viral hepatitis. Misdiagnosis or delayed recognition can result in rapid maternal and fetal deterioration. Early suspicion, timely diagnosis, and expedited delivery have transformed outcomes over the past few decades in reducing maternal and perinatal mortality.

AFLP is associated with multi-organ dysfunction. Hepatic complications include jaundice, coagulopathy, hypoglycaemia, and fulminant liver failure. Systemic complications frequently reported are acute kidney injury, disseminated intravascular coagulation, pancreatitis, sepsis, and encephalopathy. Obstetric complications include postpartum haemorrhage and preterm delivery, while fetal complications range from intrauterine growth restriction and hypoglycaemia to intrauterine death. Intensive care admission is often required, and in rare cases liver transplantation may be necessary when hepatic failure does not reverse after delivery.

Despite its severity, prognosis has improved dramatically with heightened clinical awareness and advances in perinatal and critical care. Maternal survival rates now improve especially in tertiary centres with timely diagnosis and intervention. Fetal prognosis is closely tied to early delivery, usually by induction or caesarean section depending on maternal and fetal stability. Fetal prognosis remains more guarded, with outcomes influenced by gestational age at diagnosis, intrauterine compromise, and neonatal complications of prematurity.

AFLP represents a rare but critical complication of pregnancy, demanding rapid recognition and multidisciplinary management. Its importance is underscored not only by its acute threat to maternal and fetal survival but also by its link to underlying metabolic disorders with intergenerational implications. Literature over the recent few years demonstrates significant progress in survival outcomes, yet ongoing challenges include improving diagnostic accuracy, clarifying genetic risk, and standardizing management strategies across diverse healthcare settings. Continued research into early biomarkers, targeted therapies, and global epidemiological patterns will be vital for further improving prognosis and reducing preventable maternal and perinatal morbidity and mortality.

## Introduction and background

Acute fatty liver of pregnancy (AFLP) is an uncommon yet potentially life-threatening condition that arises most often in the late stages of pregnancy or shortly after delivery. AFLP resulting in hepatic failure is a medical and obstetric emergency. Incidence varies from 1:7000 to 1:20,000 pregnancies [1]. This disorder carries substantial risks for both the mother and the fetus. Timely recognition and rapid management are essential; however, early diagnosis is often difficult due to the vague and non-specific symptoms that initially present.

Because of its clinical importance, a comprehensive literature search was conducted using databases including PubMed, Scopus, Web of Science, and Google Scholar, focusing on studies published between 2000 and 2025. Keywords such as Acute Fatty Liver in Pregnancy, AFLP, pregnancy-related liver disease, Swansea Criteria and Liver Failure in Pregnancy were used in various combinations. Inclusion criteria encompassed original studies, systematic reviews, clinical guidelines, and case series addressing the epidemiology, pathogenesis, diagnosis and management of AFLP. Articles not published in English, those focusing on non-pregnancy-related liver conditions, and those lacking full-text access were excluded.

AFLP often manifests with vague symptoms including nausea, vomiting, headache, anorexia, and abdominal discomfort. In some cases, the condition can progress quickly to severe liver dysfunction, resulting in hepatic encephalopathy, jaundice, hypoglycaemia, and coagulopathy. While symptoms typically appear during the third trimester, instances have also been documented in the late second trimester or shortly after childbirth [2].

It is important to be able to distinguish AFLP from preeclampsia/eclampsia and haemolysis, elevated liver enzymes level and low platelet counts (HELLP) as they share a similar clinical course and immediate delivery is indicated in AFLP. AFLP and HELLP can present with similar symptoms, including elevated transaminases, preeclampsia/eclampsia, and proteinuria, especially inthe third trimester. If progression to acute liver failure (ALF) occurs, liver transplant must be considered. Maternal mortality rates with AFLP can be decreased due to rapid delivery [3].

The Swansea Criteria are employed to standardize the diagnosis of AFLP. These criteria demonstrate an estimated sensitivity of 100% and a specificity of 57%, with a positive predictive value of 85% and a negative predictive value of 100%. Their accuracy was confirmed through a prospective validation study [4,5].

Management involves prompt recognition and urgent delivery, as this is the definitive treatment. Supportive care is crucial and includes stabilizing maternal hemodynamics, correcting coagulopathy, managing hypoglycaemia, and monitoring for renal and hepatic dysfunction. In severe cases, interventions such as plasma exchange or dialysis may be necessary. Multidisciplinary care is essential, involving obstetricians, intensivists, hepatologists, and neonatologists to optimize both maternal and fetal outcomes [6].

This article aims to provide an updated and comprehensive review of acute fatty liver of pregnancy, focusing on its pathogenesis, diagnostic approaches, and management strategies. It is a narrative review based on current literature to guide clinical practice and improve maternal and fetal outcomes. 

## Review

Epidemiology

AFLP is a rare but serious condition that typically arises in the third trimester or immediately postpartum [7,8]. A study analysing 12 cases of AFLP over a two-year period (2023-2024) at Maternité Souissi Hospital highlighted the importance of early diagnosis and prompt intervention in managing this condition [9]. These figures underscore the rarity of AFLP, emphasizing the need for heightened awareness and timely management to improve maternal and fetal outcomes.

AFLP is a potentially life-threatening complication. The condition most commonly affects primiparous women and is more frequently associated with a male fetus. Higher-risk populations include women with multiple gestations, previous AFLP, low body mass index (BMI), as well as those carrying fetuses with fatty acid oxidation defects, particularly long-chain 3-hydroxyacyl-CoA dehydrogenase (LCHAD) deficiency, which increases maternal susceptibility. While AFLP remains rare, maternal and perinatal morbidity can be significant, especially in resource-limited settings where delayed diagnosis is common. These epidemiologic and demographic patterns underscore the need for heightened clinical vigilance and early recognition in at-risk populations to improve outcomes [10,11].

Pathogenesis

Recent studies have elucidated the pathogenesis of AFLP, highlighting its association with mitochondrial dysfunction and impaired fatty acid oxidation.

AFLP is closely associated with fetal fatty acid oxidation defects, particularly LCHAD deficiency. When the fetus is homozygous for LCHAD deficiency, mitochondrial β-oxidation of fatty acids is impaired, leading to the accumulation of long-chain 3-hydroxy fatty acids that enter maternal circulation. Maternal factors, including heterozygous LCHAD status, increased fatty acid production during pregnancy, placental release of toxic metabolites, and reduced maternal β-oxidation, particularly in the third trimester, further contribute to AFLP pathogenesis. Accumulation of fatty acids in hepatocyte mitochondria triggers microvesicular steatosis and reactive oxygen species formation, causing maternal hepatotoxicity. Postnatally, infants with LCHAD deficiency may develop severe hypoglycaemia, myopathies, or multiorgan failure. Given this strong fetal-maternal link, it is crucial to screen infants born to mothers with AFLP for LCHAD deficiency to enable early diagnosis and intervention, preventing potentially life-threatening complications [3-12].

The placenta plays a central role in the pathogenesis of AFLP. Impaired placental mitochondrial β-oxidation redirects fatty acids toward peroxisomal oxidation, generating free radicals and releasing toxic metabolites, such as elevated arachidonic acid, into maternal circulation. This creates a harmful environment that promotes microvesicular liver steatosis, a hallmark of mitochondrial hepatopathy. Placental mitochondrial dysfunction is therefore thought to induce hepatic mitochondrial impairment, leading to acute liver failure in AFLP. Clinical observations support this mechanism, as maternal liver function often improves rapidly following delivery of the fetus and placenta, highlighting their role in disease onset [13].

AFLP arises from a complex interplay of fetal and maternal fatty acid oxidation defects, with LCHAD deficiency playing a key role. Placental mitochondrial dysfunction amplifies hepatotoxicity by generating toxic metabolites that induce maternal liver injury. Timely delivery mitigates these effects, emphasizing the central role of the fetus and placenta in disease pathogenesis.

Clinical features and complications

AFLP typically presents between 28 and 36 weeks of gestation, with a median gestational age of 36 weeks. Interestingly, up to 26% of cases may be diagnosed postpartum. Clinical features are variable and nonspecific, often overlapping with conditions such as HELLP syndrome. Gastrointestinal symptoms like nausea and vomiting are the most common, occurring in approximately 70% of cases, followed by abdominal pain (30-70%) and fatigue or polydipsia (around 30%). As the disease progresses, multi-organ involvement manifests, including hypoglycaemia, hepatic encephalopathy, severe hypoproteinaemia, ascites, gastrointestinal bleeding, and features of acute pancreatitis. Severe cases may develop acute renal failure, acute respiratory distress syndrome, pulmonary oedema, disseminated intravascular coagulation, and multi-organ failure [14]. Rising blood ammonia levels contribute to hepatic encephalopathy, causing confusion, disorientation, sleep disturbances, asterixis, and coma.

Therefore, it is important to differentiate AFLP from other hepatic disorders in pregnancy, since management and outcomes differ. Table 1 summarizes the key differences in clinical presentation, laboratory findings and management among AFLP and other common liver diseases in pregnancy [15,16]. 

**Table 1:** Comparison of differences between AFLP and other liver diseases in pregnancy [15,16] ALT: alanine aminotransferase, AST: aspartate aminotransferase, DIC: disseminated intravascular coagulation, AFLP: acute fatty liver of pregnancy, HELLP: haemolysis, elevated liver enzymes and low platelets

Feature	AFLP	HELLP	Viral Hepatitis
Onset	Late 2nd-3rd trimester, post partum	3rd trimester, usually with pre-eclampsia	Any gestation
Key Symptoms	Nausea, vomiting, abdominal pain, malaise	Right upper quadrant pain, maliase, hypertension	Jaundice, nausea, malaise
Lab Hallmarks	Hypoglycaemia, caogulopathy, mildly raised ALT/AST, raised bilirubin	Haemolysis, throbocytopenia, raised ALT/AST	Markedly raised ALT/AST, normal glucose
Complications	Encephalopathy, renal failure, DIC, multiorgan failure	Rare severe liver failure	Usually mild, rarely acute liver failure
Management	Urgent delivery + supportive care	Stabilize mother and delivery	Supportive care, treat infection

The clinical course of AFLP is typically rapid, with progression to acute liver failure over one to two weeks, marked by worsening jaundice, coagulopathy, hypoglycaemia, ascites, and encephalopathy. Delayed delivery or referral is associated with a multi-organ failure rate of approximately 38.6%. Prompt delivery usually results in rapid maternal recovery, with clinical and laboratory abnormalities returning to normal within five to seven days postpartum, highlighting the critical importance of early recognition and management [15].

Diagnosis

The Swansea Criteria are widely used to diagnose AFLP. A score of 6 or more out of 14 points, based on clinical and laboratory findings, supports the diagnosis. Key criteria include gastrointestinal symptoms (nausea/vomiting), abdominal pain, encephalopathy, elevated bilirubin, leucocytosis, elevated liver enzymes such as aspartate transaminase/ alanine transaminase (AST/ALT), elevated creatinine, prolonged prothrombin time (PT)/activated partial thromboplastin time (APTT), ultrasound findings (hepatic steatosis). A score of 6 or more is considered diagnostic, though histopathological confirmation via liver biopsy is rarely performed due to its invasive nature during pregnancy [8-17].

AFLP must be distinguished from other liver disorders in pregnancy, such as HELLP syndrome and viral hepatitis. While HELLP syndrome presents with haemolysis, elevated liver enzymes, and low platelets, AFLP is characterized by hypoglycaemia, hyperbilirubinemia, and coagulopathy without significant haemolysis. Viral hepatitis typically presents with markedly elevated liver enzymes and normal glucose levels, differing from the biochemical profile seen in AFLP [15,16].

Prompt recognition and diagnosis of AFLP are critical due to its potential for rapid progression to liver failure. Utilizing the Swansea Criteria facilitates early identification, enabling timely intervention and improving maternal and fetal outcomes.

Management

Management of AFLP requires a multidisciplinary approach involving obstetricians, hepatologists, intensivists, and neonatologists. The mainstay of therapy is prompt delivery, alongside intensive supportive care to prevent maternal and fetal complications. Early recognition and intervention are critical, as delays significantly increase maternal and neonatal morbidity and mortality.

Prompt Delivery

Immediate delivery is the primary treatment for AFLP, as it halts disease progression and allows maternal recovery within five to seven days postpartum [15]. Vaginal delivery is preferred if rapid progression is feasible, but caesarean section is indicated in cases of fetal or maternal distress, unripe cervix, or inability to deliver quickly. Prior to delivery, correction of hypoglycaemia, careful fluid-electrolyte balance, correction of coagulation abnormalities with blood products such as fresh frozen plasma, cryoprecipitate, fibrinogen, and platelets is essential. Preventive measures against postpartum haemorrhage should also be implemented immediately after birth to minimize complications and optimize maternal outcomes [15,18].

In AFLP patients, a multidisciplinary rapid response team should be established preoperatively to plan anaesthesia and surgical management. Coagulation status is the key determinant for anaesthesia choice. Patients with International Normalised Ratio (INR) ≤1.2 are suitable for intraspinal anaesthesia, while those with INR between 1.2 and 1.5 may undergo single spinal or local nerve block anaesthesia. General anaesthesia is recommended for patients with INR ≥1.5 or unstable hemodynamic. This individualized approach ensures maternal safety, reduces anaesthesia-related risks, and optimizes surgical outcomes in critically ill AFLP patients requiring urgent delivery [19,20].

Management of Complications and Acute Liver Failure

Plasma exchange in AFLP may improve oxidative stress, apoptosis markers, hepatic recovery, and reduce intensive care unit (ICU) stay, though mortality benefit remains unproven. When combined with plasma perfusion or continuous renal replacement therapy in severe cases with multiorgan dysfunction, it may enhance clinical outcomes, though evidence is limited by small retrospective studies [21,22].

Hepatic encephalopathy is a common complication of acute fatty liver of pregnancy, ranging from mild confusion to severe intracranial hypertension and cerebral oedema, both major contributors to morbidity and mortality. Unlike in chronic liver disease, lactulose and rifaximin are ineffective in acute liver failure [23]. N-acetylcysteine, known for its benefits in paracetamol toxicity, may improve outcomes in non-drug-induced acute liver failure, including AFLP, with minimal adverse effects, and is therefore considered in moderate to severe cases [23,24]. Nonconvulsive seizures may occur with severe liver failure, but there is no evidence supporting prophylactic antiepileptic use in these patients [25].

Cerebral oedema and intracranial hypertension can develop rapidly in acute liver failure, a complication of AFLP, and significantly worsen prognosis. Intracranial pressure (ICP) monitoring is the gold standard, with recommended targets of ICP <20 mmHg and cerebral perfusion pressure >50 mmHg [26]. Management strategies include elevating the head of the bed to 30°, using propofol sedation in intubated patients, avoiding hypercapnia, and administering hypertonic saline (200 ml of 2.7% sodium chloride (NaCl) or 20 ml of 30% NaCl) or mannitol. Therapy should be titrated to maintain blood osmolality below 320 mOsm/L, aiming to reduce ICP and prevent neurological deterioration [27-29].

Infections are frequent in acute liver failure, including AFLP, affecting sites such as the lungs, urinary tract, bloodstream, and surgical wounds. Routine prophylactic antibiotics are not advised, but broad-spectrum antibiotics should be initiated if hypotension worsens or encephalopathy progresses. While prophylaxis does not improve survival, regular surveillance cultures and a low threshold for starting antibiotics or antifungals are recommended, given the high risk and potential morbidity of infection in these patients [26,30].

In acute liver failure associated with ALFP, empiric correction of coagulopathy with vitamin K is recommended, while blood products and antifibrinolytic agents should be administered based on bleeding severity, lab abnormalities, and clinical judgment, as no definitive transfusion threshold exists [31]. Prognostic evaluation relies on coagulation status, so aggressive transfusion without active haemorrhage is discouraged. Additionally, nutritional support, either enteral or parenteral, is essential to manage hypoglycaemia and support the increased catabolic demands associated with acute liver failure [23].

In severe cases of acute liver failure, including AFLP, intubation and mechanical ventilation, fluid resuscitation, stress dose of steroids for relative adrenal insufficiency may be required to support respiratory function, optimize oxygenation, and facilitate overall patient care. Renal replacement therapy is often necessary for patients with acute kidney injury or fluid-electrolyte imbalances, which are common complications. Management of metabolic derangements and electrolyte abnormalities can be particularly challenging due to the interplay between hepatic, renal, and systemic dysfunction. Close monitoring in an intensive care setting, along with individualized interventions, is essential to stabilize organ function, prevent further complications, and optimize maternal outcomes [32].

Liver Transplant 

Liver transplantation (LT) is a critical intervention for patients with AFLP who progress to acute liver failure despite optimal supportive care and timely delivery. Indications include persistent coagulopathy, encephalopathy, renal failure, and refractory metabolic disturbances despite maximal supportive care.

A retrospective study utilizing the U.S. Scientific Registry of Transplant Recipients data from 1991 to 2015 identified women undergoing LT for AFLP-related acute liver failure. These patients often presented with severe illness, including renal dysfunction and hyperbilirubinemia, and had comparable early and late survival outcomes to other acute liver failure aetiologies. However, cumulative five-year graft survival was numerically lower in the AFLP group (54%) compared to acetaminophen-induced (70%) and other causes (76%) of acute liver failure. This difference may be attributed to higher rates of graft rejection in AFLP patients [33]. 

Given the rarity of AFLP, early referral to a transplant centre is essential for timely assessment and listing. Multidisciplinary coordination ensures optimal perioperative management, including correction of coagulopathy and stabilization of organ function prior to surgery [15].

In conclusion, while liver transplantation is a life-saving option for AFLP patients with severe liver failure, outcomes may be less favourable compared to other acute liver failure causes. Early recognition, prompt delivery, and timely referral to transplant centres are crucial for improving maternal and fetal outcomes.

## Conclusions

AFLP is a rare, potentially life-threatening disorder, typically arising in the third trimester or postpartum. Rapid progression to acute liver failure and multi-organ dysfunction makes early recognition and multidisciplinary management essential. The primary treatment is prompt delivery, supported by intensive care addressing hypoglycaemia, coagulopathy, renal failure, and hepatic encephalopathy. In severe cases unresponsive to conventional therapy, liver transplantation offers a lifesaving option, with generally favourable maternal outcomes, though graft survival may be slightly lower than in other acute liver failure aetiologies due to higher rejection rates. Early referral to transplant centres, careful perioperative preparation, and coordination between obstetric, hepatology, and critical care teams are crucial.

This article provides significant insights into AFLP’s pathophysiology, diagnosis, and management, emphasizing timely intervention and multidisciplinary care. It highlights supportive strategies for managing complications and clarifies the role of transplantation in severe cases. By comparing AFLP with other causes of acute liver failure, the article guides clinicians on risk assessment, early referral, and perioperative management. Overall, it serves as a comprehensive resource, improving understanding of disease progression, refining treatment strategies, and contributing to better maternal and fetal outcomes in this rare but serious condition.
